# Effect of preserved eggs on the health of SD rats, and anti‐tumor action of HT‐29 cells

**DOI:** 10.1002/fsn3.3558

**Published:** 2023-07-26

**Authors:** Yan Wu, Changyi Mao, Gan Hu, Lulu Ma, Shugang Li, Meihu Ma

**Affiliations:** ^1^ National Research and Development Center for Egg Processing, College of Food Science and Technology Huazhong Agricultural University Wuhan People's Republic of China; ^2^ School of Food and Biological Engineering Hefei University of Technology Hefei People's Republic of China

**Keywords:** active component, anti‐tumor, health, preserved eggs

## Abstract

Preserved eggs are traditional alkali‐pickled food in China and have been enjoyed by consumers and extensively studied by researchers for their nutritional tastes and their anti‐tumor, anti‐inflammatory, antioxidant, lipid‐lowering, and blood pressure‐lowering properties. To study the anti‐tumor effects of preserved eggs, this project observed the health on rats, and anti‐tumor effects and separated anti‐tumor active components on HT‐29 cells. SD rats fed for 80 days showed that preserved eggs had no significant effect on weight, food intake, blood pH, liver tissues, or organ indices. Preserved eggs significantly increased blood levels of oxidative stress markers SOD and CAT, decreased MDA levels by 0.46, 0.23, and 0.25 times. Moreover, they also increased the level of IL‐2 from 1233 to 1340 pg/mL. Two water‐soluble bioactive peptide fractions, B_1_ and B_2_, with molecular weights ≥10 kDa were further obtained from preserved eggs by ultrafiltration and Superdex Peptide 10/300 GL. The potential mechanism of B_1_ and B_2_ is to activate the internal mitochondrial apoptotic pathway and induce apoptosis by up‐regulating the expression of the pro‐apoptotic factors cytochrome C, caspase‐3, and caspase‐9 mRNA in HT‐29 cells.

## Graphical abstract

Our study demonstrated that preserved eggs did not affect normal physiological functions in rats, whose dietary behavior, body pH, and organ indices remained normal. It also had significant antioxidant effects. Two water‐soluble fractions, B_1_ and B_2_, with MW ≥10 kDa, were screened from preserved eggs. Their anti‐tumor mechanism was to induce apoptosis in HT‐29 cells by up‐regulating the expression of pro‐apoptotic factors CytC, caspase‐3 and caspase‐9 mRNA.

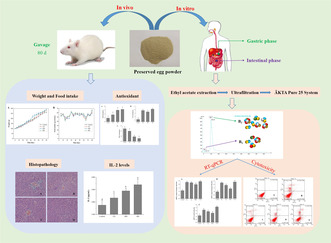

## 1 INTRODUCTION

Preserved eggs are a type of traditional Chinese alkaline pickled food favored by many people for their unique flavors (Zhang et al., 2018). In the production of preserved eggs, fresh duck eggs which are rich in proteins, such as oval‐transferrin, ovalbumin, ovomucin, and ovomucoid, are treated with alkaline (Batool, Hu, Xinyue, et al., 2021; Ge et al., 2021). It has been exported to different countries around the world, such as Korea, Japan, Thailand, and Malaysia (Xu et al., 2013; Zhao et al., 2016).

The preserved eggs have shown multi‐functions including lowering blood lipids, regulating intestinal flora, antioxidant, anti‐hypertensive, anti‐inflammatory, and anti‐tumor activity (Mao et al., 2018; Meng et al., 2020; Zhao et al., 2016). Preserved egg digestions (PED) are usually composed of peptides, amino acids, and proteins (Zhang et al., 2019). They not only down‐regulated gene expression of pro‐inflammatory cytokines in the colon, including tumor necrosis factor‐alpha (TNF‐α), interleukin‐6 (IL‐6), and interleukin‐1β (IL‐1β) (Zhao et al., 2017) but also significantly inhibited the secretion of pro‐inflammatory factors including nitric oxide (NO), TNF‐α and IL‐6 (Ding et al., 2019). It has also been found that PED promoted apoptosis in HT‐29 and HepG2 cells, (Liang et al., 2020). The PED have been reported to induce apoptosis in renal carcinoma cells by up‐regulating the expression of pro‐apoptotic factors Bax and interleukin‐10 (IL‐10), and down‐regulating the expression of anti‐apoptotic factors interleukin‐4 (IL‐4) and IL‐6 (Batool, Hu, Huang, et al., 2021). In a recent study, Preserved eggs were reported to cause a dramatic increase in reactive oxygen species (ROS) in HepG2 cells. At the same time, they activated the mitochondria‐mediated apoptotic pathway by upregulating the expression of the pro‐apoptotic gene Bcl‐2 and inhibiting the expression of the anti‐apoptotic gene Bak. In addition, they reduced the expression of the angiogenic factors Hypoxia‐inducible factor‐1α (HIF‐1α) and Vascular endothelial growth factor (VEGF), which may lead to inhibition of cell growth and migration (Wu et al., 2023). The peptides DR‐10 and DF‐9 isolated from PED can exert anti‐inflammatory effects by inhibiting NF‐κB and MAPK signaling pathways (Zhao et al., 2017). The peptides smaller than 5 kDa and isolated from preserved egg white reduced the secretion of the anti‐inflammatory markers TNF‐α and IL‐8 (Zhang et al., 2020; Zhao et al., 2017). However, the health effects and anti‐tumor active components are still unclear and further studies are needed.

To observe the effect of preserved eggs feeding on rats and screen for the anti‐tumor active fractions, in vitro and in vivo experiments were performed on Sprague–Dawley (SD) rats and colon cancer cells (HT‐29). After 80 days of preserved egg feeding, body weight, food intake, organ index and liver tissue of rats were measured. Moreover, its anti‐tumor active fraction was initially isolated and screened. This provides a theoretical basis for the further promotion and high‐value utilization of preserved eggs and also lays the foundation for the screening of anti‐tumor active peptides of preserved eggs.

## 2 MATERIALS AND METHODS

### 2.1 Materials and reagents

Pepsin, trypsin, the McCoy's 5A Medium, and fetal bovine serum (FBS) were purchased from Sigma‐Aldrich, St. Louis, USA. The penicillin/streptomycin was obtained from Nacalai Tesque Inc., Kyoto, Japan. The cell counting kit‐8 (CCK‐8), 5‐Fluorouracil (5‐FU) and hematoxylin/eosin (HE) were provided by Shanghai Yuanye Bio‐Technology Co., Ltd. (Shanghai, China). The IL‐2, caspase‐3, caspase‐9, and cytochrome C (CytC) kit were purchased from Beyotime Institute of Biotechnology (Shanghai, China). All other chemicals were purchased from Sinopharm Chemical Reagent Co., Ltd (Shanghai, China).

### 2.2 In vivo study

#### 2.2.1 Preparation of preserved egg powders

The duck eggs were purchased from Hubei Shendan Health Food Co., Ltd. Preserved eggs were obtained by following the modified method of (Zhao et al., 2014). Fresh intact duck eggs (50–70 g) were selected and soaked in 4.5% NaOH, 3% NaCl, 0.4% CuSO_4_, and 3.5% Chinese black tea at 25°C for about 5 weeks. Then, they were washed, peeled, homogenized at 10,000 rpm for 30 s by a homogenizer (HG‐200, Hsiangtai Machinery Industry Co., Ltd) and then lyophilized using a lyophilizer (Alphal‐4LSC, Martin Christ Gefriertrocknungsanlagen Co., Ltd., Germany). Afterwards, the lyophilized powders was stored at 4°C, for a maximum of 2 weeks. The water content of the powder was analyzed according to China National Standards CNS GB 5009.3–2010.

#### 2.2.2 Animals and experimental design

With the animal care and use protocol approved by the Institutional Animal Care and Use Committee of Huazhong Agricultural University, the study was performed following the Regulations for the Administration of Affairs Concerning Experimental Animals. (Animal Welfare No. 00140424, Animal Quality Certificate No. 42000600014606).

The female Sprague Dawley rats aged 2–3 weeks (100.00 ± 0.13 g) were purchased from Tongji Medical College, Huazhong Science and Technology University. All the rats (*n* = 40) were housed and treated according to the requirements of the Laboratory Animal Ethics Review Committee. The animals were allowed to take normal rat feed and water (20–25 g/day). A 12 h light/12 h dark room lighting cycle was used in the room. The room temperature and relative humidity were controlled within the range of 25 ± 2°C and 50 ± 5%, respectively. After a seven‐day acclimation period, eight rats were sacrificed by decannulation as 0‐day group. The remaining rats were randomly divided into 4 groups (*n* = 8 per group), including one control group and three dosed groups: low‐dose group (LD, 1.4 g/100 g), middle‐dose group (MD, 2.8 g/100 g), and high‐dose group (HD, 4.2 g/100 g) as detailed in Figure S.1. Each rat was gavaged with 2 mL/day for 80 days. During the experiment, the body weight and food intake of each rat were measured every 5 days.

#### 2.2.3 Blood pH, IL‐2 level and visceral indexes

Rat blood was taken from the retro‐orbital plexus, then blood pH and IL‐2 levels were measured in rats using a blood gas analyzer and IL‐2 kit, respectively. The rat spleen, kidney, liver, and perisplenic adipose were weighed, and the visceral indexes were calculated using following equation.

(1) $$\text{Visceral index}\;\left(\%\right)=W_{i,l,j,k}/W_{0}\times 100$$

where *W*_*i,l,j,k*_ is the visceral weight (*i, l, j, k* are spleen, kidney, liver, and perisplenic adipose weights of rats, respectively), g; and *W*_*0*_ is weight of rats, g.

### 2.3 Analysis of oxidative stress markers

According to the ratio of liver tissue weight (g): volume (mL) = 1: 9, nine times volume of 0.9% saline was added to liver tissue and tissues were homogenized in an ice‐water bath. Then, the tissue was centrifuged at 2500 rpm for 10 min at 4°C and the supernatant was taken for determination. Then, the activities of oxidative stress markers, catalase (CAT), malondialdehyde (MDA), and superoxide dismutase (SOD) in the supernatant were determined using colorimetric microplate assay according to the instructions (Nanjing Jiancheng Institute of Biological Engineering, Nanjing, China) by using a microplate reader (iMark, Lenovo Biological Technology Co., Ltd, China). The CAT, MDA, and SOD activity were expressed as mmol g^−1^ protein.

### 2.4 Histopathological analysis

Fresh liver tissues were fixed in 4% paraformaldehyde solution for more than 24 h. Subsequently, the tissues were embedded in paraffin and cut to a thickness of 4 μm by a rotary machine, followed by HE staining. The histopathological images of the liver were taken under a microscope.

### 2.5 In vitro anti‐tumor active ingredient screening

#### 2.5.1 Cell culture

HT‐29 cells were purchased from the cell bank of the Chinese Academy of Sciences (Shanghai, China). HT‐29 cells were cultured using the Tao et al. method (Tao et al., 2021) as described in Supporting information S.1.

#### 2.5.2 Preparation of PED

The PED preparation procedure was executed by Liang's method with a slight modification (Liang et al., 2020) as highlighted in Supporting information S.2.

#### 2.5.3 Separation of PED

The water‐soluble and lipid‐soluble components in the PED were separated by ethyl acetate (EtOAc) extraction. 400 mg PED were mixed with 50 mL distilled water and 16 mL ethyl acetate. The mixture was transferred to a separating funnel and gently shaken to mix the two phases. It is then left to stand and once the two phases were completely separated, slowly rotated the piston to release the lower liquid, and then poured out the upper liquid from the separating funnel. The ethyl acetate in the extraction product was removed using a rotary evaporator (RE‐52AA, Shyarong, Shanghai, China). The water‐soluble and lipid‐soluble resultants were lyophilized and stored at 4°C for subsequent studies.

#### 2.5.4 Primary screening of active ingredients

The HT‐29 cells were seeded in a 96‐well plate at a density of 5 × 10^4^ cells/well, and incubated in 100 μL McCoy's 5A medium for 24 h. Then, the cells were treated with 100 μL/well of water‐soluble and lipid‐soluble components, respectively, 5‐FU (2 mg/mL) were used as positive control. Ten microliter CCK‐8 solution was added to each well and incubated for 2 h, the absorbance was then measured at 450 nm using a microplate reader. The morphology of the cells was observed by fluorescence microscopy (OLYMPUS IX71, Tokyo, Japan). The components with the best effect to inhibit the proliferation of HT‐29 cells were selected and recorded as component A. The following equation was used to calculate the cell proliferation inhibition rate (CPIR)

(2) $$\text{CPIR}\;\left(\%\right)=\frac{\left(A-B\right)}{A}\times 100$$

where *A* and *B* are the OD_450_ of the control and sample, respectively.

#### 2.5.5 Separation and screening of component A

The component A was separated using 3 and 10 kDa ultrafiltration tubes. During the separation, the component A was mixed with sterile water at a ratio of 1:1 (w/v) and added to ultrafiltration centrifuge tubes with a cut‐off MW of 3 kDa by centrifugation at 4000 rpm for 40 min at 4°C (Sigma3‐30 k, Sigma Aldrich, St, Louis, USA). The ≥3 kDa fraction was then added to a 10 kDa centrifuge tube, centrifuged at 4000 rpm for 40 min at 4°C, and repeated twice. The three components were collected and marked as A_1_ (MW <3 kDa), A_2_ (3 kDa ≤ MW < 10 kDa), and A_3_ (MW ≥10 kDa). The inhibitory effects of A_1_, A_2_, and A_3_ on HT‐29 tumor cells were determined by the CCK‐8 method. The component having the best effects among A_1_, A_2_,and A_3_ was then marked as component B.

### 2.6 Purification of component B

Component B was separated using Superdex peptide 10/300GL GE gel filtration columns via the ÄKTA™ pure 25 system (Agilent Technologies, Inc.). The component B was dissolved in deionized water at a concentration of 20 mg/mL, filtered through a 0.22 μm microporous membrane, and then injected into the machine with a 2 mL injection volume. The elution was performed at a flow rate of 0.5 mL/min using deionized water as the mobile phase, and meanwhile, the components with different peaks were collected using 280 nm as the detection line. Under this condition, the elution was repeated several times for bulk preparation. The elution peak sample solutions were collected and lyophilized, then label as B_1_ and B_2_ and store at −20°C.

#### 2.6.1 RT‐qPCR analysis

The genes involved in cell apoptosis metabolism were analyzed by quantitative reverse transcription‐polymerase chain reaction (RT‐qPCR) (Zhong et al., 2018), as underlined in Supporting information S.3.

#### 2.6.2 Cellular apoptosis assay

Apoptosis was measured by Annexin V‐FITC/PI (Zoman Biotechnology Co., Ltd. Beijing, China) double‐staining method (Ma et al., 2019), as detailed in Supporting information S.4.

### 2.7 Statistics and analysis

One‐way ANOVA was performed for each experiment, and statistical significance was obtained by using Duncan's multiple range test on IBM SPSS Statistics 20.0 (SPSS Inc, Chicago, IL, USA), where *p* < .05 was considered to be significant.

## 3 RESULTS AND DISCUSSION

### 3.1 Effect of preserved eggs on body weight and food intake

**FIGURE 1:**
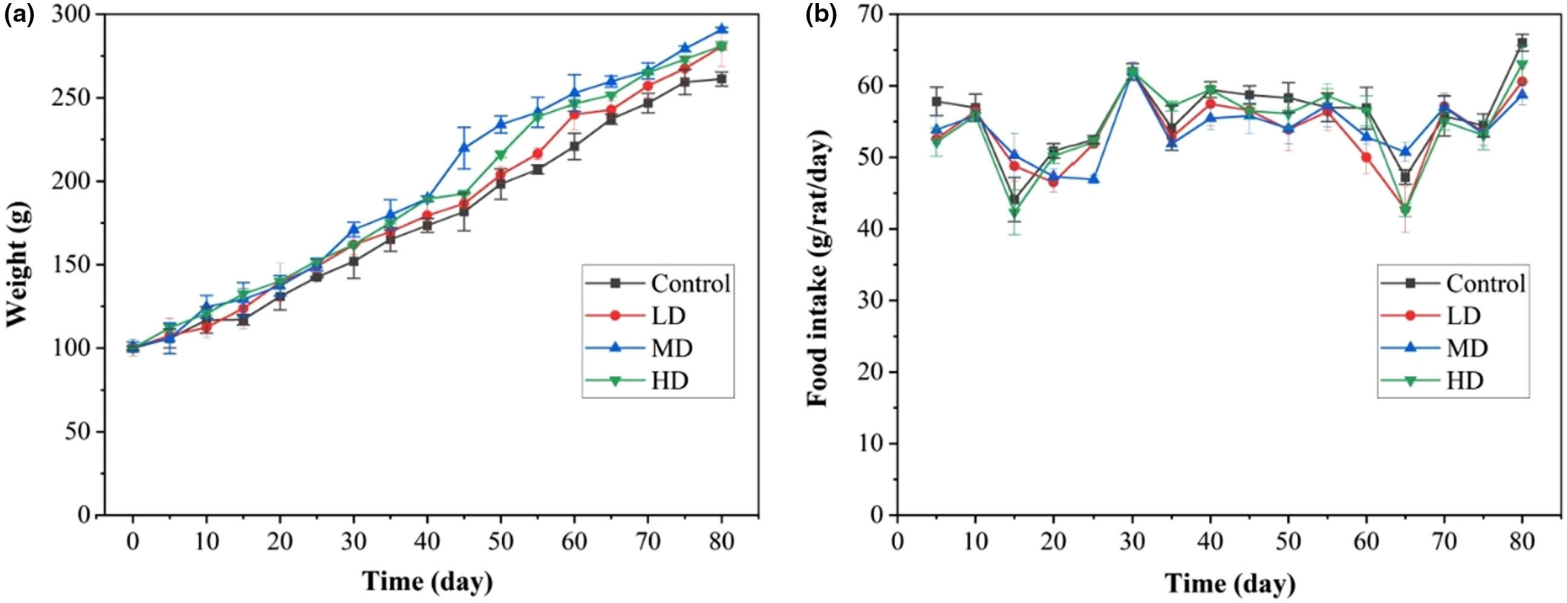
Effects of preserved eggs on body weight (A) and food intake (B) of rats. Control: 0 g/100 g; LD: low‐dose group, 1.4 g/100 g; MD: middle‐dose group, 2.8 g/100 g; HD: high‐dose group, 4.2 g/100 g.

SD rats are fast‐growing and have reliable breeding performance, so they are widely used in safety and nutrition‐related tests. In pathophysiology, overweight or marked weight loss is considered as an indicator of health problems (Poirier et al., 2006). As shown in Figure 1A, after 80 days of feeding with PED (water content of 1.32%), the rats gained weight normally from 100.00 to 290.71 g, not significantly different from the control group (*p* > .05). Furthermore, the daily intake of each rat was between 42.31 and 66.00 g after feeding with preserved eggs (Figure 1B), and the significance analysis showed no significant difference in the intake of rats (*p* > .05). Therefore, preserved eggs did not adversely affect the eating behavior of rats. Table 1 shows the effect of preserved eggs on the blood pH of rats. After 80 days of feeding, the blood pH still remained in the normal range of 7.00–7.40 (Zausinger et al., 2002) and no statistical difference (*p* > .05) was found. This suggested that long‐term intake of preserved eggs did not affect the eating behavior of the rats or the acid–base balance of their blood.

**TABLE 1:** Changes in blood pH of rats fed preserved eggs (*n* = 8).

Time/day	Control	LD	MD	HD
0	7.23 ± 0.01^a^	7.23 ± 0.01^a^	7.23 ± 0.01^a^	7.23 ± 0.01^a^
80	7.26 ± 0.01 ^a^	7.23 ± 0.02^a^	7.24 ± 0.01^a^	7.18 ± 0.02^a^

*Note*: Same letters in the same column indicate no significant difference in pH between 0 and 80 days (*p* > .05). Control: 0 g/100 g; LD: low‐dose group, 1.4 g/100 g; MD: middle‐dose group, 2.8 g/100 g; HD: high‐dose group, 4.2 g/100 g.

### 3.2 Changes in visceral indexes

The viscera index refers to the ratio of organ weight to body weight and is a direct assessment of the extent of lesions in the internal organs (Yan et al., 2021). Table 2 showed that after 80 days of preserved eggs intervention, the visceral indexes of spleen, kidney, liver, and perisplenic adipose in each group were not significantly different compared with the control group (*p* > .05), indicating that long‐term intake of preserved eggs did not affect the normal growth of organs in SD rats. In the current animal studies, it has been demonstrated that preserved eggs significantly reduced triglyceride and total cholesterol levels, as well as low‐density lipoprotein (LDL‐C)/high‐density lipoprotein (HDL‐C) levels in the liver of rats, which is related to lipid metabolism in rats (van der Made et al., 2014).

**TABLE 2:** Changes in visceral indexes of rats fed preserved eggs (*n* = 8).

Group	Spleen index	Kidney index	Liver index	Perisplenic adipose index
Control	0.22 ± 0.03^a^	0.62 ± 0.05^b^	2.77 ± 0.18^c^	0.28 ± 0.02^d^
LD	0.22 ± 0.01^a^	0.64 ± 0.03^b^	2.74 ± 0.28^c^	0.27 ± 0.02^d^
MD	0.23 ± 0.04^a^	0.65 ± 0.04^b^	2.88 ± 0.25^c^	0.33 ± 0.04^d^
HD	0.32 ± 0.18^a^	0.64 ± 0.03^b^	2.72 ± 0.18^c^	0.27 ± 0.01^d^

*Note*: Same letters in the same column indicate no significant difference in the visceral indexes for LD,MD and HD compared with the control group (*p* > .05).

### 3.3 Effects of preserved eggs on the CAT, MDA, and SOD values

The three primary antioxidant enzymes, namely CAT, SOD, and GPx contained in the cells were thought to be necessary for life in all oxygen metabolizing cells (Bertero et al., 2019). MDA is an important indicator of membrane system injuries and deterioration of cellular metabolism caused by environmental stress (Fan et al., 2012). SOD and CAT can play an antioxidant role by quenching free radicals and inhibiting the production of harmful products such as MDA (Shi et al., 2021). Furthermore, the SOD, CAT, and MDA are key to redox homeostasis in the gastrointestinal body (Bertero et al., 2019).

**FIGURE 2:**
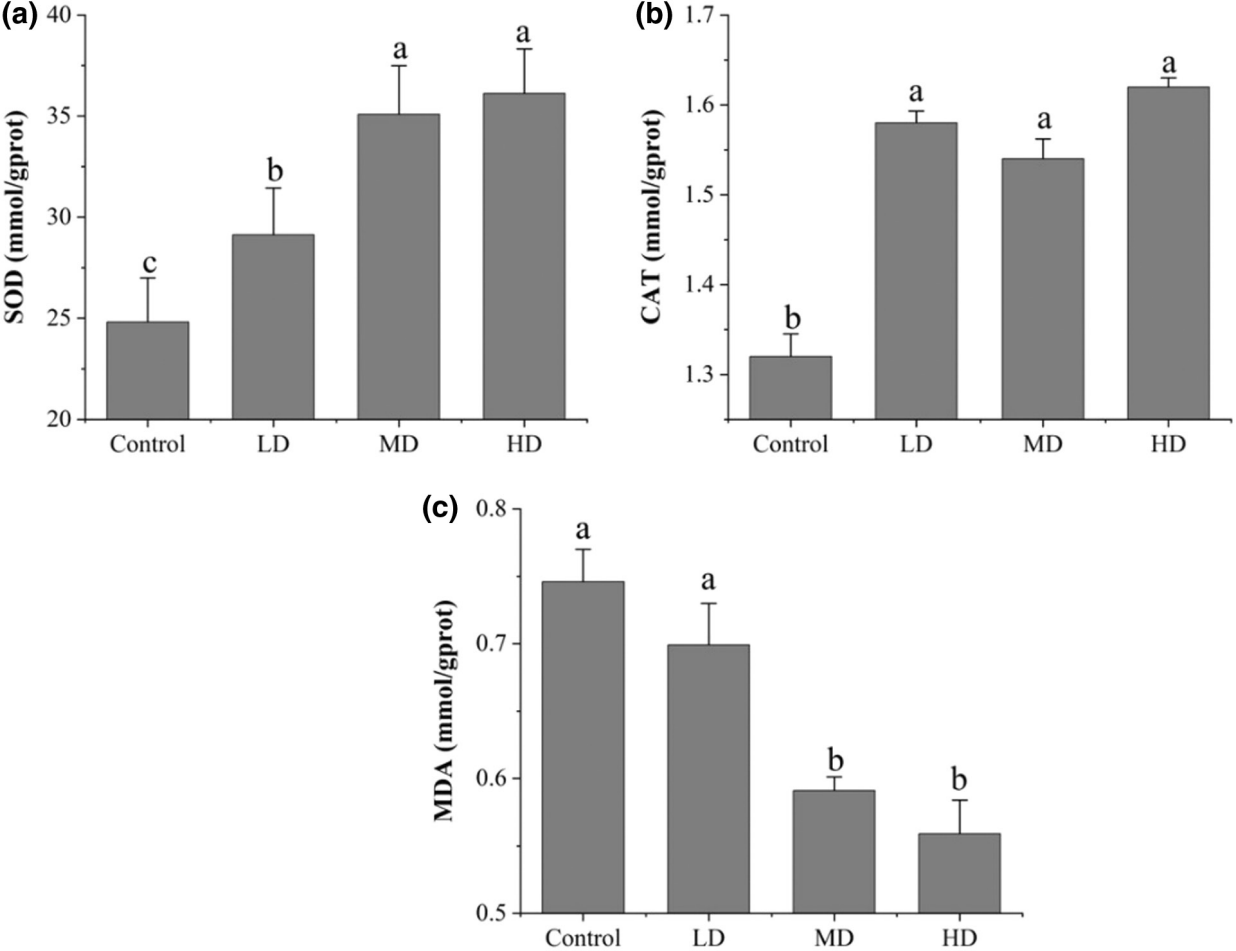
Effects of preserved eggs on oxidation indicators of rats. (a) SOD; (b) CAT; (c) MDA. The letters in the figure indicate significant differences (*p* < .05).

Figure 2 shows the changes in SOD, CAT and MDA in the liver of rats. The liver SOD activity was increased (*p* < .05) and dose‐dependent after treatment, and the HD group was approximately 1.5 times larger than the control group. Meanwhile, CAT levels were increased (*p* < .05) to about 1.2 times of the control group. However, there was no significant difference in the CAT expression between LD, MD, and HD groups (*p* > .05). Furthermore, MDA levels decreased consistently in a dose‐dependent manner from 0.699 to 0.559 mmol/gprot. These results suggested that preserved eggs exhibited their antioxidant activity by increasing the levels of SOD and CAT activities and decreasing the levels of MDA.

### 3.4 Effect of preserved eggs on the histopathology of liver tissue

**FIGURE 3:**
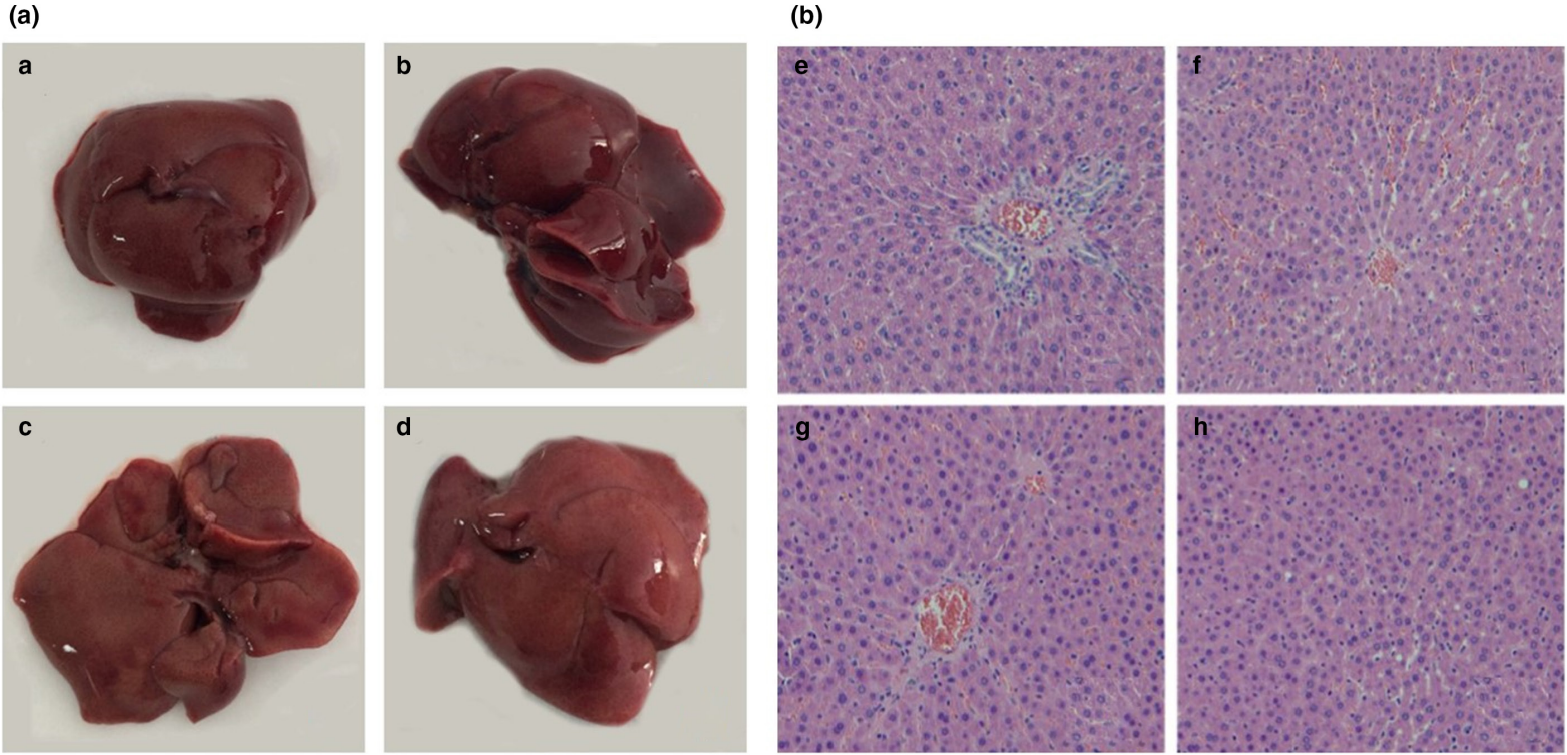
Liver dissection (A) and histopathological findings (HE, 100×) (B). (a, e) Control; (b, f) LD; (c, g) MD; (d, h) HD.

The liver is an organ with mainly metabolic functions and has functions such as de‐oxidation, storage of liver sugar, and secretory protein synthesis (Boby et al., 2021). Figure 3A shows the liver tissue anatomy of each group. The liver tissue of rats in the control group was bright red in color, smooth on the surface, soft and elastic in texture without visible particles. There was no significant difference in the treatment group compared to the control group.

The histopathological results of the liver are shown in Figure 3B. The control group showed no obvious abnormalities under light microscopy, with normal cell size, neat arrangement, clear margins, and intact nuclei morphology. In the meantime, the cell plasma was evenly stained, and no inflammatory cells were observed. No significant difference was found among the LD group, the MD group, and the control group. A small number of round fat vacuoles appeared in the HP group, but they did not reach the extent of liver lesions, and no inflammatory cell infiltration and hepatocyte necrosis were observed. Therefore, long‐term intake of preserved eggs did not lead to liver tissue damage or risk of fatty liver disease.

### 3.5 Effect of preserved eggs on IL‐2 content

**FIGURE 4:**
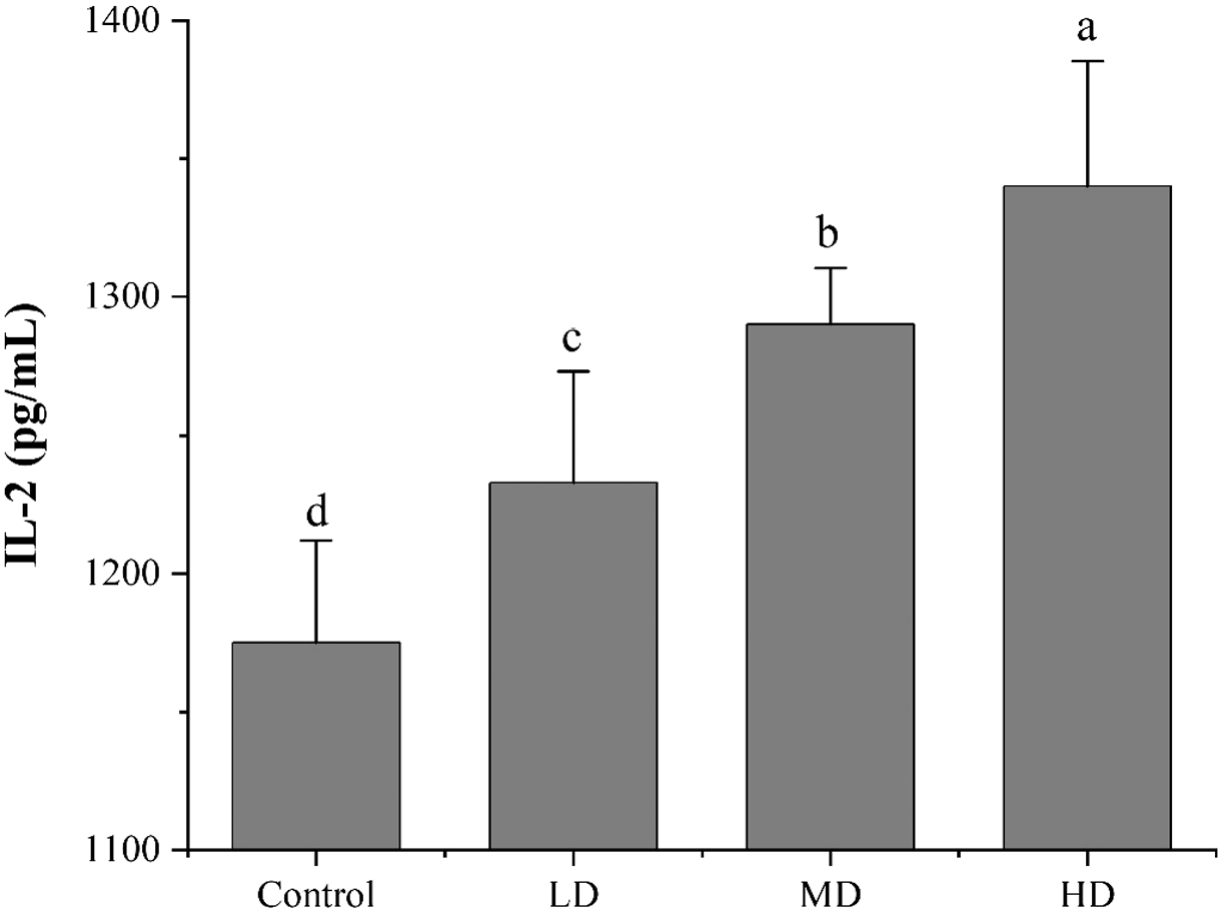
Effects of preserved eggs on IL‐2 in blood of rats in 80 days. The letters in the figure indicate significant differences (*p* < .05).

IL‐2 induces biological effects in lymphokine‐activated killer cells (LAK) and cytotoxic CD8 T lymphocytes (CTL), enhancing their ability to lyse tumor cells, and has been found in numerous studies to play an important role in anti‐tumor (Abbas et al., 2018). Figure 4 shows the changes of IL‐2 in serum after 80 days of feeding. As shown in the figure, the level of IL‐2 in the control group was 1175 pg/mL. In the LD, MD, and HD groups, the intake of preserved egg significantly increased the expression of IL‐2 in rat serum, from 1233 pg/mL to 1290 pg/mL and 1340 pg/mL (*p* < .05), in a dose‐dependent manner. This indicates that the preserved eggs can increase the expression of IL‐2 in rat serum within a certain dose range and exert anti‐tumor effects. A recent study confirmed that preserved eggs induced apoptosis in kidney cancer cells Caki‐1, ACHN and 786‐O. The possible mechanism was through upregulation of the levels of the pro‐apoptotic genes Bax and IL‐10 and reduction in the expression of the anti‐apoptotic factors IL‐4 and IL‐6 (Batool, Hu, Huang, et al., 2021).

### 3.6 The effects of lipid‐ and water‐soluble components on HT‐29 cells

**FIGURE 5:**
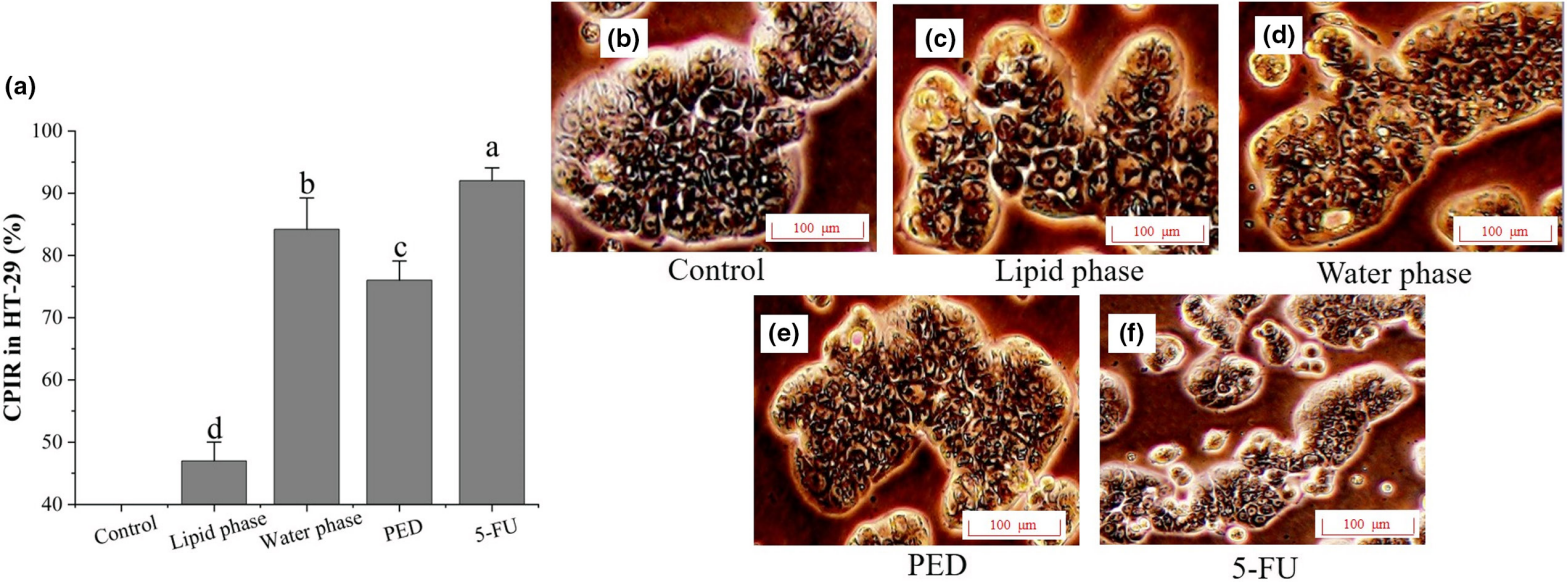
The effects of lipid‐ and water‐soluble on CPIR (a) and morphological changes (B‐F) of HT‐29 cells. The letters in figure A indicate significant differences (*p* < .05).

To explore the anti‐tumor mechanism, the effects of lipid‐soluble and water‐soluble components on the CPIR and morphology of HT‐29 cells were studied. As shown in Figure 5A, the control group had the smallest CPIR of 0.20%, while the values of the lipid‐soluble and water‐soluble fractions were 47.00% and 84.20%, respectively. The water‐soluble fraction was less effective than the positive anti‐tumor drug 5‐FU (92%) in inhibiting the growth of HT‐29 cells. Morphology in Figure 5B showed that untreated cells displayed a normal polygonal structure without significant damage. The cells treated with the lipid‐soluble components underwent slight changes with bleaching of the cell membrane and chromatin condensation (Figure 5C), while those treated with water‐soluble components were easily observed with blurred cell edges, disorganized cell structure, and disappearance of visible nuclei (Figure 5D). Therefore, the water‐soluble component which disrupted the structure of tumor cells and inhibited their growth and proliferation was chosen to investigate the effects on HT‐29 cell apoptosis.

### 3.7 Effect of different molecules of component a on HT‐29 cells

It was found that HT‐29 cells activity was significantly inhibited at a PED concentration of 2 mg/mL. Simultaneously, HT‐29 cells underwent contraction, plasma membrane blistering, and chromatin condensation associated with DNA cleavage into steps. In addition, PED induced apoptosis, with the apoptosis rate of HT‐29 cells in the high concentration group being 16.5%, which was 5.8 times higher than that of the control group (Liang et al., 2020). This suggested that PED could effectively promote apoptosis in HT‐29 tumor cells, but the exact mechanism needs to be further investigated.

**FIGURE 6:**
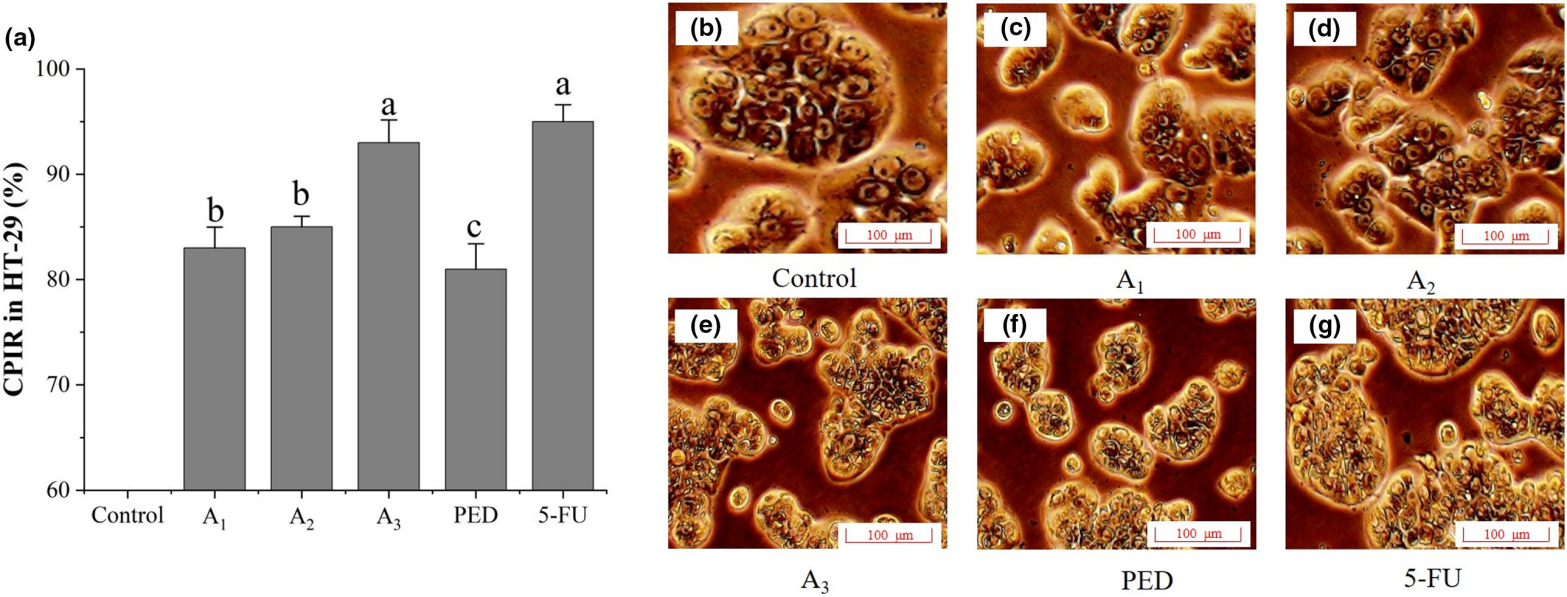
The effect of different molecules of component A on CPIR (A) and morphological changes (B–G) of cells. The letters in figure A indicate significant differences (*P* < 0.05).

Figure 6A compares the effects of different fractions on CPIR of HT‐29 cells. The CPIRs of A_1_, A_2_ and PED on HT‐29 cells were 83%, 85%, and 81%, respectively. A_3_ had the highest CPIR of 90% and was not significantly different (*p* > .05) from the 5‐FU group (95%). This indicated that A_3_ had the best tumor suppression effect. Figure 6B–G compares the morphological changes of HT‐29 cells in different treatments. The commonly known apoptosis characteristics are cell distortion, cell shrinkage, cell membrane blebbing, and chromatin condensation, which may be linked with cleavage of DNA (Lee et al., 2002). Untreated cells in the control were tightly arranged with a normal polygonal structure. Compared with A_1_ and A_2_, which had little effect on the HT‐29 cell structure, the cells in A_3_ were mostly distorted with defocused cell edges and disappearance of nuclei, indicating cell death due to apoptosis. Therefore, A_3_ was selected as component B.

### 3.8 The ÄKTA™ pure system analysis component B

**FIGURE 7:**
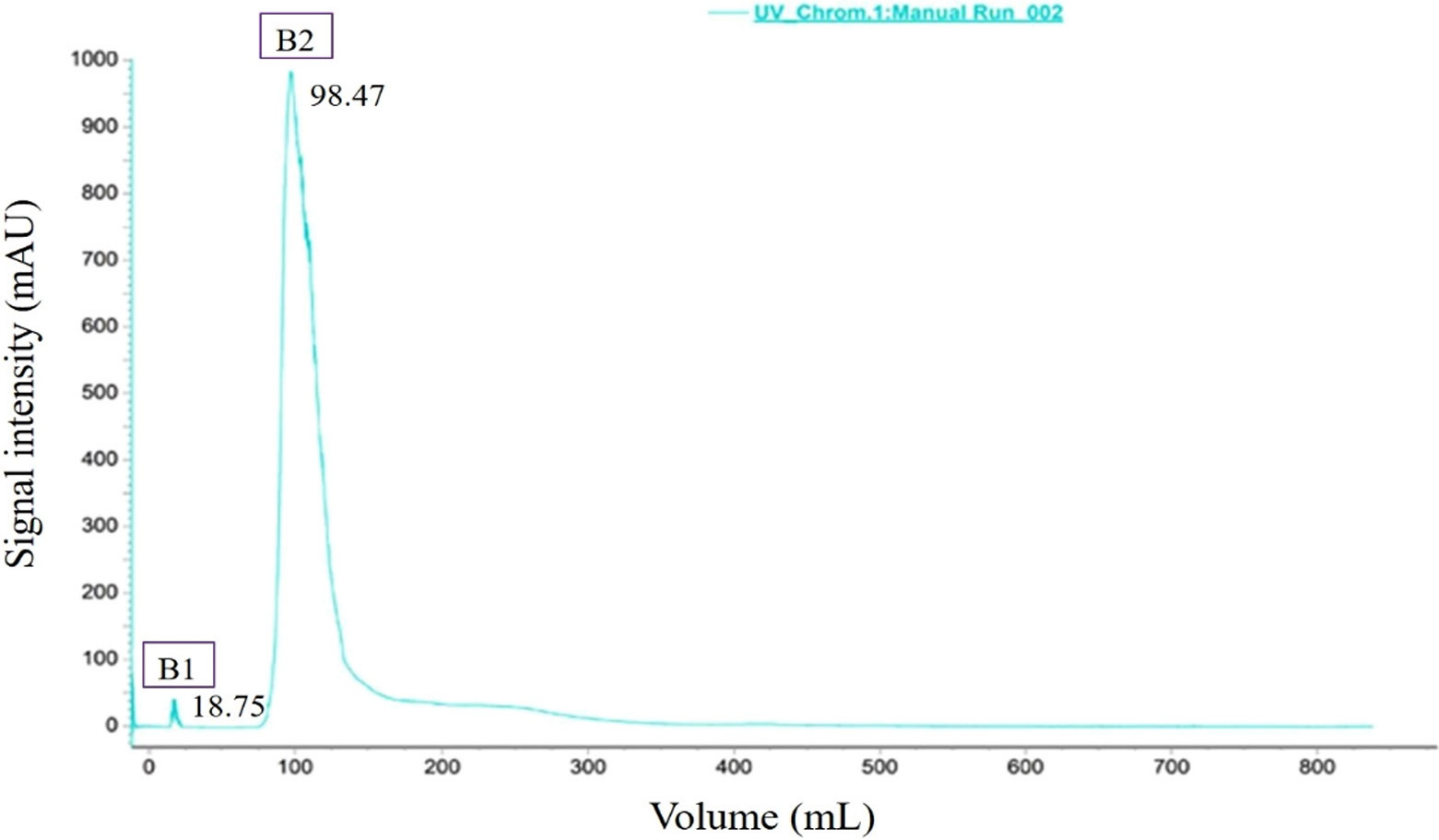
Chromatogram of gel filtration for component B by ÄKTA Pure 25 using Superdex 200 10/300 GL.

The separation of component B by gel chromatography is shown in Figure 7, where two peaks with MW ≥10 kDa were found and named B_1_ and B_2_. The basic principle of gel chromatography, also known as molecular sieving, is to separate substances according to their molecular size by the distribution of pore sizes in gel molecular sieves (Worsztynowicz et al., 2020). Therefore, B_2_ had a smaller MW and significantly higher content than B_1_ (*p* < .05). Although the ÄKTA™ Pure 25 System allows easy and fast differentiation of the peptide fractions, it executes preliminary separation and purification. Better purification can be achieved by combining different methods, such as solid‐phase metal affinity chromatography IMAC coupled with mass spectrometry (MALDI‐TOF‐MS) (Gagnaire et al., 2009). Further studies should focus on the analytical identification of B_1_ and B_2_ to determine their molecular weight and specific structure.

### 3.9 Effect of B_1_ and B_2_ on Caspase‐3, Caspase‐9, and CytC mRNA expression

Caspase‐3 and caspase‐9 are important regulatory proteases in the mitochondrial pathway, with caspase‐3 mainly involved in the late stages of apoptosis (Jeon et al., 2017; Micucci et al., 2015; Wang et al., 2017). CytC, located in the outer mitochondrial membrane and mediates cellular mitochondrial apoptosis, plays an important role in mitochondrial energy metabolism by performing electron transfer between respiratory chain complex enzyme III and respiratory chain complex enzyme IV (Liu et al., 2007). When apoptotic signals reach the mitochondria, pro‐ and anti‐apoptotic proteins of the Bcl family can act together to release CytC, which then activates caspase‐9 by forming an apoptotic complex and eventually activates caspase‐3 to induce apoptosis (Shao et al., 2018).

**FIGURE 8:**
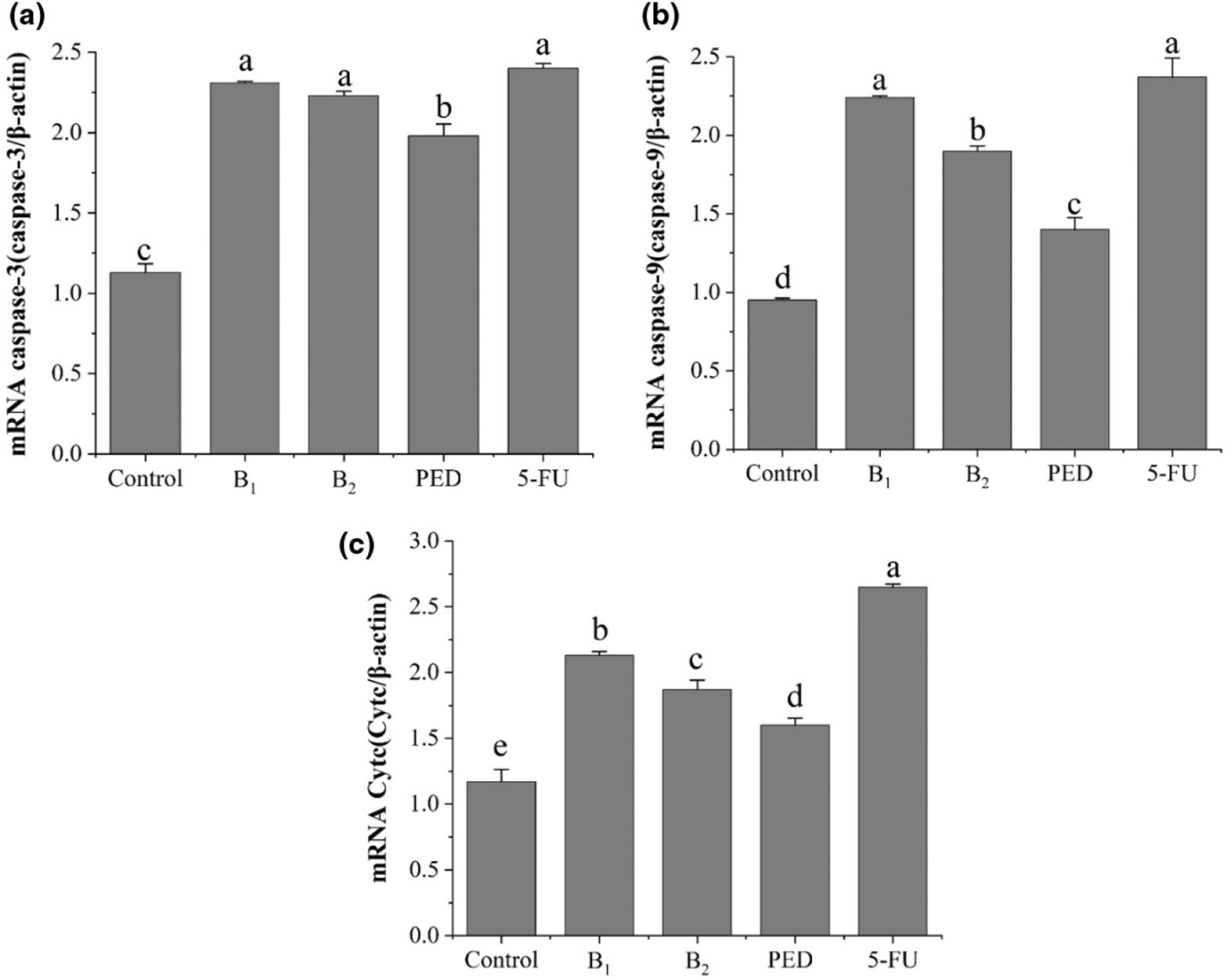
The expression of gene mRNA exposed to B_1_ and B_2_ in HT‐29 cells. (A) Caspase‐3; (B) Caspase‐9; (C) CytC. The letters in the figures indicate significant differences (*p* < .05).

It was reported that PED can induce apoptosis in HT‐29 and HepG2 cells by upregulating the expression of Bax mRNA and downregulating the expression of Bcl‐2 mRNA and COX‐2 mRNA to promote the release of caspase‐3 and caspase‐9 (Liang et al., 2020). As shown in Figure 8, compared with the control group, B_1_ up‐regulated the expressions of caspase‐3 caspase‐9 and CytC to 1.04, 1.36, and 0.82 times, and B_2_ up‐regulated those expressions to 0.97, 1.00, and 0.60 times, respectively (*p* < .05). The expressions in PED were superior to that of the control but less than those in both B_1_ and B_2_.

### 3.10 Effect of B_1_ and B_2_ on apoptosis

**FIGURE 9:**
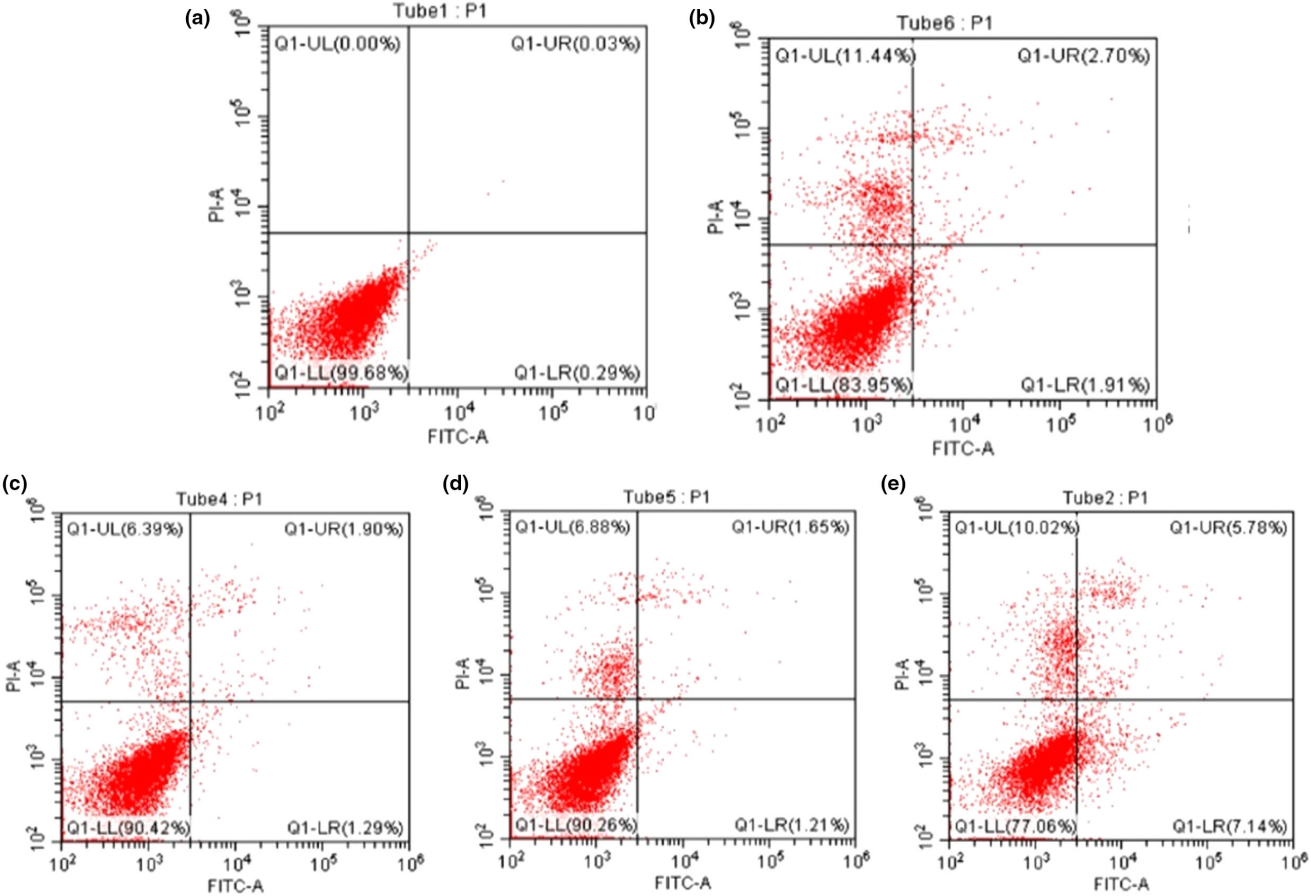
The effect of B_1_ and B_2_ on apoptosis of HT‐29 cells. (A) Control; (B): B_1;_ (C): B_2;_ (D) PED; (E) 5‐FU.

The effects of B_1_ and B_2_ on the apoptosis of HT‐29 cells are shown in Table 3 and Figure 9, where the early and late apoptosis characterized by DNA fragmentation after activation of the caspase pathway were in the lower right and upper right corners, respectively. The apoptosis rates of HT‐29 cells in B_1_ and B_2_ were 4.61% and 3.19% respectively, slightly higher than 2.86% in the PED group, and much higher than 0.32% in the control group (*P* < 0.05). Therefore, we supposed that the anti‐tumor effect was achieved through peptide fractions action and was closely related to the mitochondrial pathway (So et al., 2009).

**TABLE 3:** Cell apoptosis in HT‐29 cells exposed to B_1_ and B_2_.

Sample	Control	B_1_	B_2_	PED	5‐FU
Apoptosis rate (%)	0.32^d^	4.61^b^	3.19^c^	2.86^c^	12.92^a^

*Note*: Cell apoptosis in HT‐29 cells exposed to B_1_ and B_2_ were significantly different compared with the control group (*p* < .05).

## 4 CONCLUSIONS

Our study demonstrated that preserved eggs did not affect normal physiological functions in rats, whose dietary behavior, body pH, and organ indices remained normal. Preserved eggs protected hepatocytes from damage by increasing the antioxidant capacity of tissues through up‐regulating SOD and CAT expression, and decreasing MDA expression. Preserved eggs also significantly up‐regulated IL‐2 expression, showing anti‐tumor effects. Two water‐soluble fractions, B_1_ and B_2_, with MW ≥10 kDa, were screened from preserved eggs. Their anti‐tumor mechanism was to induce apoptosis in HT‐29 cells by up‐regulating the expression of pro‐apoptotic factors CytC, caspase‐3, and caspase‐9 mRNA. This suggested that B_1_ and B_2_ could lead to the release of CytC, which activated caspases to cause apoptosis and achieved the purpose of promoting tumor cell apoptosis through the mitochondrial pathway. In the present study, two anti‐tumor fractions, B_1_ and B_2_, were isolated from the preserved eggs. However, the anti‐tumor mechanism of the preserved eggs, and they anti‐tumor fractions need to be further studied. More research on the anti‐tumor properties of the preserved eggs should be focused on in vivo experiments.

## AUTHOR CONTRIBUTIONS

**Yan Wu:** Conceptualization (equal); data curation (equal); formal analysis (equal); methodology (equal); visualization (equal); writing – original draft (equal); writing – review and editing (equal). **Changyi Mao:** Data curation (equal); formal analysis (equal); methodology (equal); writing – review and editing (equal). **Gan Hu:** Visualization (equal); writing – review and editing (equal). **Lulu Ma:** Investigation (equal); supervision (equal); writing – review and editing (equal). **Shugang Li:** Methodology (equal); supervision (equal). **Meihu Ma:** Supervision (equal); writing – review and editing (equal).

## CONFLICT OF INTEREST STATEMENT

The authors declared that they have no conflicts of interest to this work. We declare that we do not have any commercial or associative interest that represents a conflict of interest in connection with the work submitted.

## ETHICS STATEMENT

This study has been approved for animal ethics.

## Supporting information


Data S1.

## Data Availability

Data sharing not applicable to this article as no data‐sets were generated or analyzed during the current study.

## References

[fsn33558-bib-0001] Abbas, A. K. , Trotta, E. , Simeonov, D. , Marson, A. , & Bluestone, J. A. (2018). Revisiting IL‐2: Biology and therapeutic prospects. Science Immunology, 3, eaat1482. 10.1126/sciimmunol.aat1482 29980618

[fsn33558-bib-0002] Batool, Z. , Hu, G. , Huang, X. , Wu, Y. , Fu, X. , Cai, Z. , Huang, X. , & Ma, M. (2021). Dietary therapeutic treatment of renal carcinoma cell lines by down‐regulating cFlip, Mcl‐1, Bcl‐XL and STAT3 gene expression under the influence of up‐regulated Bax and intrinsic apoptotic pathway. Food Bioscience, 43, 101319. 10.1016/j.fbio.2021.101319

[fsn33558-bib-0003] Batool, Z. , Hu, G. , Xinyue, H. , Wu, Y. , Fu, X. , Cai, Z. , Huang, X. , & Ma, M. (2021). A comprehensive review on functional properties of preserved eggs as an excellent food ingredient with anti‐inflammatory and anti‐cancer aspects. Food Bioscience, 44, 101347. 10.1016/j.fbio.2021.101347

[fsn33558-bib-0004] Bertero, T. , Oldham, W. M. , Grasset, E. M. , Bourget, I. , Boulter, E. , Pisano, S. , Hofman, P. , Bellvert, F. , Meneguzzi, G. , Bulavin, D. V. , Estrach, S. , Feral, C. C. , Chan, S. Y. , Bozec, A. , & Gaggioli, C. (2019). Tumor‐stroma mechanics coordinate amino acid availability to sustain tumor growth and malignancy. Cell Metabolism, 29, 124–140. 10.1016/j.cmet.2018.09.012 30293773 PMC6432652

[fsn33558-bib-0005] Boby, N. , Lee, E.‐B. , Abbas, M. A. , Park, N. H. , Lee, S. P. , Ali, M. S. , Lee, S. J. , & Park, S. C. (2021). Ethanol‐induced hepatotoxicity and alcohol metabolism regulation by GABA‐enriched fermented *Smilax China* root extract in rats. Food, 10, 2381. 10.3390/foods10102381 PMC8535858 34681429

[fsn33558-bib-0006] Ding, N. , Mao, C. , Cai, Z. , & Ma, M. (2019). Anti‐inflammatory effect of preserved egg with simulated gastrointestinal digestion on LPS‐stimulated RAW264. 7 cells. Poultry Science, 98, 4401–4407. 10.3382/ps/pez243 31041437

[fsn33558-bib-0034] Fan, W. , Zhang, M. , Zhang, H. , & Zhang, P. (2012). Improved tolerance to various abiotic stresses in transgenic sweet potato (ipomoea batatas) expressing spinach betaine aldehyde dehydrogenase. PLoS ONE, 7(5), e37344. 10.1371/journal.pone.0037344 22615986 PMC3353933

[fsn33558-bib-0007] Gagnaire, V. , Jardin, J. , Jan, G. , & Lortal, S. (2009). Invited review: Proteomics of milk and bacteria used in fermented dairy products: From qualitative to quantitative advances. Journal of Dairy Science, 92, 811–825. 10.3168/jds.2008-1476 19233774

[fsn33558-bib-0008] Ge, S. , Ferreira Júnior, Á. , Zhang, X. , & Morgan, P. M. (2021). The domestic hen. In X. Y. Zhang , R. S. Vieira‐Pires , P. M. Morgan , & R. Schade (Eds.), IgY‐technology: Production and application of egg yolk antibodies (pp. 15–20). Springer Nature Switzerland AG.

[fsn33558-bib-0009] Jeon, M.‐Y. , Min, K.‐j. , Woo, S. M. , Seo, S. U. , Kim, S. , Park, J. W. , & Kwon, T. K. (2017). Volasertib enhances sensitivity to TRAIL in renal carcinoma Caki cells through downregulation of c‐FLIP expression. International Journal of Molecular Sciences, 18, 2568. 10.3390/ijms18122568 29186071 PMC5751171

[fsn33558-bib-0010] Lee, W.‐R. , Shen, S.‐C. , Lin, H.‐Y. , Hou, W. C. , Yang, L. L. , & Chen, Y. C. (2002). Wogonin and fisetin induce apoptosis in human promyeloleukemic cells, accompanied by a decrease of reactive oxygen species, and activation of caspase 3 and Ca^2+^‐dependent endonuclease. Biochemical Pharmacology, 63, 225–236. 10.1016/S0006-2952(01)00876-0 11841797

[fsn33558-bib-0011] Liang, Y. , He, L. , Zhang, M. , Liu, X. , Jin, G. , Jin, Y. , & Ma, M. (2020). Preserved egg digests promote the apoptosis of HT29 and HepG2 cells. Food Bioscience, 36, 100661. 10.1016/j.fbio.2020.100661

[fsn33558-bib-0012] Liu, L. , Peng, J. , Liu, K. , Yang, H. , Li, Y. , & Hong, H. (2007). Influence of cytochrome c on apoptosis induced by *Anagrapha* (*Syngrapha*) falcifera multiple nuclear polyhedrosis virus (AfMNPV) in insect *Spodoptera litura* cells. Cell Biology International, 31, 996–1001. 10.1016/j.cellbi.2007.03.011 17478109

[fsn33558-bib-0013] Ma, Y.‐L. , Zhang, Y.‐S. , Zhang, F. , Zhang, Y.‐Y. , Thakur, K. , Zhang, J.‐G. , & Wei, Z.‐J. (2019). Methyl protodioscin from *Polygonatum sibiricum* inhibits cervical cancer through cell cycle arrest and apoptosis induction. Food and Chemical Toxicology, 132, 110655. 10.1016/j.fct.2019.110655 31271762

[fsn33558-bib-0014] Mao, C. , Yu, Z. , Li, C. , Jin, Y. , & Ma, M. (2018). The functional properties of preserved eggs: From anti‐cancer and anti‐inflammatory aspects. Korean Journal of Food Science and Technology, 38, 615–628. 10.5851/kosfa.2018.38.3.615 PMC6048375 30018504

[fsn33558-bib-0015] Meng, Y. , Chen, C. , Qiu, N. , & Keast, R. (2020). Modulation of gut microbiota in rats fed whole egg diets by processing duck egg to preserved egg. Journal of Bioscience and Bioengineering, 130, 54–62. 10.1016/j.jbiosc.2020.02.015 32224011

[fsn33558-bib-0016] Micucci, C. , Matacchione, G. , Valli, D. , Orciari, S. , & Catalano, A. (2015). HIF2α is involved in the expansion of CXCR4‐positive cancer stem‐like cells in renal cell carcinoma. British Journal of Cancer, 113, 1178–1185. 10.1038/bjc.2015.338 26439684 PMC4647880

[fsn33558-bib-0035] Poirier, P. , Giles, T. D. , Bray, G. A. , Hong, Y. , Stern, J. S. , Pi‐Sunyer, F. X. , & Eckel, R. H. (2006). Obesity and cardiovascular disease: Pathophysiology, evaluation, and effect of weight loss. Circulation, 113(6), 898–918. 10.1161/circulationaha.106.171016 16380542

[fsn33558-bib-0017] Shao, X. , Chen, Q. , Dou, X. , Chen, L. , Wu, J. , Zhang, W. , Shao, H. , Ling, P. , Liu, F. , & Wang, F. (2018). Lower range of MW of xanthan gum inhibits cartilage matrix destruction via intrinsic bax‐mitochondria cytochrome c‐caspase pathway. Carbohydrate Polymers, 198, 354–363. 10.1016/j.carbpol.2018.06.108 30093011

[fsn33558-bib-0018] Shi, X. , Cheng, W. , Wang, Q. , Zhang, J. , Wang, C. , Li, M. , Zhao, D. , Wang, D. , & An, Q. (2021). Exploring the protective and reparative mechanisms of *G. lucidum* polysaccharides against H_2_O_2_‐induced oxidative stress in human skin fibroblasts. Clinical, Cosmetic and Investigational Dermatology, 14, 1481–1496. 10.2147/CCID.S334527 34703264 PMC8525518

[fsn33558-bib-0019] Tao, S. , Huang, C. , Tan, Z. , Duan, S. , Zhang, X. , Ren, Z. , Zhou, C. , Huang, J. , Liu, C. , & Wei, G. (2021). Effect of the polysaccharides derived from *Dendrobium officinale* stems on human HT‐29 colorectal cancer cells and a zebrafish model. Food Bioscience, 41, 100995. 10.1016/j.fbio.2021.100995

[fsn33558-bib-0020] van der Made, S. M. , Kelly, E. R. , Berendschot, T. T. , Kijlstra, A. , Lütjohann, D. , & Plat, J. (2014). Consuming a buttermilk drink containing lutein‐enriched egg yolk daily for 1 year increased plasma lutein but did not affect serum lipid or lipoprotein concentrations in adults with early signs of age‐related macular degeneration. The Journal of Nutrition, 144, 1370–1377. 10.3945/jn.114.195503 24991045

[fsn33558-bib-0021] Wang, Y. , Gao, W. , Shi, X. , Ding, J. , Liu, W. , He, H. , Wang, K. , & Shao, F. (2017). Chemotherapy drugs induce pyroptosis through caspase‐3 cleavage of a gasdermin. Nature, 547, 99–103. 10.1038/nature22393 28459430

[fsn33558-bib-0022] Worsztynowicz, P. , Białas, W. , & Grajek, W. (2020). Integrated approach for obtaining bioactive peptides from whey proteins hydrolysed using a new proteolytic lactic acid bacteria. Food Chemistry, 312, 126035. 10.1016/j.foodchem.2019.126035 31901822

[fsn33558-bib-0023] Wu, Y. , Li, X. , Ma, M. , Hu, G. , Fu, X. , & Liu, J. (2023). Characterization of the dynamic gastrointestinal digests of the preserved eggs and their effect and mechanism on HepG2 cells. Food, 12, 800. 10.3390/foods1204080 PMC9955911 36832875

[fsn33558-bib-0024] Xu, L. , Yan, S. M. , Cai, C. B. , Yu, X. P. , Jiang, J. H. , Wu, H. L. , & Yu, R. Q. (2013). Nonlinear multivariate calibration of shelf life of preserved eggs (Pidan) by near infrared spectroscopy: Stacked least squares support vector machine with ensemble preprocessing. Journal of Spectroscopy, 2013, 1–7. 10.1155/2013/797302

[fsn33558-bib-0025] Yan, S. , Wang, K. , Wang, X. , Ou, A. , Wang, F. , Wu, L. , & Xue, X. (2021). Effect of fermented bee pollen on metabolic syndrome in high‐fat diet‐induced mice. Food Science and Human Wellness, 10, 345–355. 10.1016/j.fshw.2021.02.026

[fsn33558-bib-0026] Zausinger, S. , Baethmann, A. , & Schmid‐Elsaesser, R. (2002). Anesthetic methods in rats determine outcome after experimental focal cerebral ischemia: Mechanical ventilation is required to obtain controlled experimental conditions. Brain Research Protocols, 2002, 112–121. 10.1016/S1385-299X(02)00138-1 12034330

[fsn33558-bib-0027] Zhang, M. , Zhao, Y. , Yao, Y. , Xu, M. , du, H. , Wu, N. , & Tu, Y. (2019). Isolation and identification of peptides from simulated gastrointestinal digestion of preserved egg white and their anti‐inflammatory activity in TNF‐α‐induced Caco‐2 cells. The Journal of Nutritional Biochemistry, 63, 44–53. 10.1016/j.jnutbio.2018.09.019 30342316

[fsn33558-bib-0028] Zhang, Y. , Liu, Y. , Yang, W. , Huang, J. , Liu, Y. , Huang, M. , Sun, B. , & Li, C. (2018). Characterization of potent aroma compounds in preserved egg yolk by gas chromatography–olfactometry, quantitative measurements, and odor activity value. Journal of Agricultural and Food Chemistry, 66, 6132–6141. 10.1021/acs.jafc.8b01378 29790747

[fsn33558-bib-0029] Zhang, Z. , Xu, T. , Qin, W. , Huang, B. , Chen, W. , Li, S. , & Li, J. (2020). Upregulated PTPN2 induced by inflammatory response or oxidative stress stimulates the progression of thyroid cancer. Biochemical and Biophysical Research Communications, 522, 21–25. 10.1016/j.bbrc.2019.11.047 31735335

[fsn33558-bib-0030] Zhao, Y. , Chen, Z. , Li, J. , Xu, M. , Shao, Y. , & Tu, Y. (2016). Changes of microstructure characteristics and intermolecular interactions of preserved egg white gel during pickling. Food Chemistry, 203, 323–330. 10.1016/j.foodchem.2016.02.044 26948621

[fsn33558-bib-0031] Zhao, Y. , Tu, Y. , Xu, M. , Li, J. , & du, H. (2014). Physicochemical and nutritional characteristics of preserved duck egg white. Poultry Science, 93, 3130–3137. 10.3382/ps.2013-03823 25332139

[fsn33558-bib-0032] Zhao, Y. , Yao, Y. , Xu, M. , Wang, S. , Wang, X. , & Tu, Y. (2017). Simulated gastrointestinal digest from preserved egg white exerts anti‐inflammatory effects on Caco‐2 cells and a mouse model of DSS‐induced colitis. Journal of Functional Foods, 35, 655–665. 10.1016/j.jff.2017.06.028

[fsn33558-bib-0033] Zhong, X.‐Y. , Yuan, X.‐M. , Xu, Y.‐Y. , Yin, M. , Yan, W. W. , Zou, S. W. , Wei, L. M. , Lu, H. J. , Wang, Y. P. , & Lei, Q. Y. (2018). CARM1 methylates GAPDH to regulate glucose metabolism and is suppressed in liver cancer. Cell Reports, 24, 3207–3223. 10.1016/j.celrep.2018.08.066 30232003

