# Correction to: “Effect of preserved eggs on the health of SD rats, and anti‐tumor action of HT‐29 cells”

**DOI:** 10.1002/fsn3.4151

**Published:** 2024-06-05

Wu, Y., Mao, C., Hu, G., Ma, L., Li, S., & Ma, M. (2023). Effect of preserved eggs on the health of SD rats, and anti‐tumor action of HT‐29 cells. *Food Science & Nutrition*, *11*, 6188–6198. https://doi.org/10.1002/fsn3.3558

In the published version of the article, there were errors in the affiliation attributions. The correct authors and affiliations appear below.

Yan Wu^1,2^, Changyi Mao^2^, Gan Hu^2^, Lulu Ma^1^, Shugang Li^1^, Meihu Ma^2^.

^1^School of Food and Biological Engineering, Hefei University of Technology, Hefei, People's Republic of China.

^2^National Research and Development Center for Egg Processing, College of Food Science and Technology, Huazhong Agricultural University, Wuhan, People's Republic of China.

The Supporting Information has also been replaced in the online version, with a corrected version of Table S1.

We apologize for this error.

