# Supplementary material for: Effect of preserved eggs on the health of SD rats, and anti‐tumor action of HT‐29 cells
Source: Food Sci Nutr. 2023 Jul 26;11(10):6188–98. doi: 10.1002/fsn3.3558 (PMC10563691; doi:10.1002/fsn3.3558)
Supplement: Supplementary file 1 — Data S1. [file FSN3-11-6188-s001.docx]

**SUPPLEMENTARY MATERIALS**

***S.1 Cell Culture***

HT-29 cells were grown in 89% McCoy’s 5A Medium and supplemented with 10% heat-inactivated FBS and 1% penicillin/streptomycin in a humidified incubator with 5% CO_2_ in air at 37˚C (Heracell™ 150i, Thermo Scientific, Germany). Cell passage numbers 10-30 in the logarithmic phase of growth were used for subsequent experiments.

***S.2 Preparation of PED***

Firstly, 20 mg of preserved egg powder was dissolved in 200 mL of distilled water and adjusted to pH 2.0 with 1 M HCl. Second, pepsin was added in a ratio of 0.125 g/10 g sample. Then, the mixture was incubated at 37℃ for 2 h, adjusted to pH 7.0 with 1 M NaHCO_3_, mixed with trypsin (0.2 g/10 g sample), and incubated at 37℃ for 4 h. After that, the resultants were kept in boiling water for 10 min to terminate the reaction, waited until cooled to room temperature, and centrifuged at 11,000×g for 15 min (9130 rpm in a JA-12 rotor, Avanti® j-26 XP, Beckman Coulter, Inc., Palo Alto, CA, USA). The final supernatant was lyophilized and stored at -20℃ for two weeks.

***S.3 RT-qPCR Analysis***

HT-29 cells were treated with different components B_1_ (0.5 mg/mL), B_2_ (0.5 mg/mL), 5-FU (2 mg/mL), and PED. HT-29 cells were harvested after 24 h of treatment. RNA of HT-29 cells was then extracted by E.Z.N.A® Total RNA Kit 1 following the user manual. The primers for PCR were designed using the Premier 5 software and synthesized by Wuhan Biofavor biotechnology Service Co., Ltd. The sequences are listed in Table S.1, where β-actin was used as the endogenous control. Each experiment was repeated 3 times independently. The data analysis was accomplished by the 2^–ΔΔCt^ method.

***S.4 Cellular Apoptosis Assay.***

Apoptosis was measured by Annexin V-FITC/PI (Zoman Biotechnology Co., Ltd. Beijing, China) double-staining method (Ma et al., 2019). HT-29 cells were inoculated at a density of 2.5×10^5^ cells/well on 6-well plates at 37°C with 5% CO_2_ and cultured overnight. After that, the original medium was removed, carefully rinsed with PBS, and then the incubation was continued by adding B_1_ and B_2_ (0.5 mg/mL), respectively, while 5-FU (2 mg/mL) was used as a positive control and culture medium was used as a negative control. After 24 h, cells were taken out and dissociated by trypsin. The cells were then washed with PBS and centrifuged at 1,000 rpm for 5 min to remove the supernatant. After that, cells were resuspended with Annexin V Binding Buffer (pH 7) and the concentration was adjusted to 1×10^6^/mL, and then 5 μL Annexin V-FITC was added at room temperature and stored in dark for 10 min. Afterwards, 10 μL PI was added and the mixture was further incubated for 5 min at room temperature and analyzed using flow cytometry (FCM, FACSCalibur, Becton Dickson, San Jose, CA, USA) within 1 h. For each sample, 2×10^4^ events were acquired. Apoptosis analysis was repeated for at least three independent experiments.


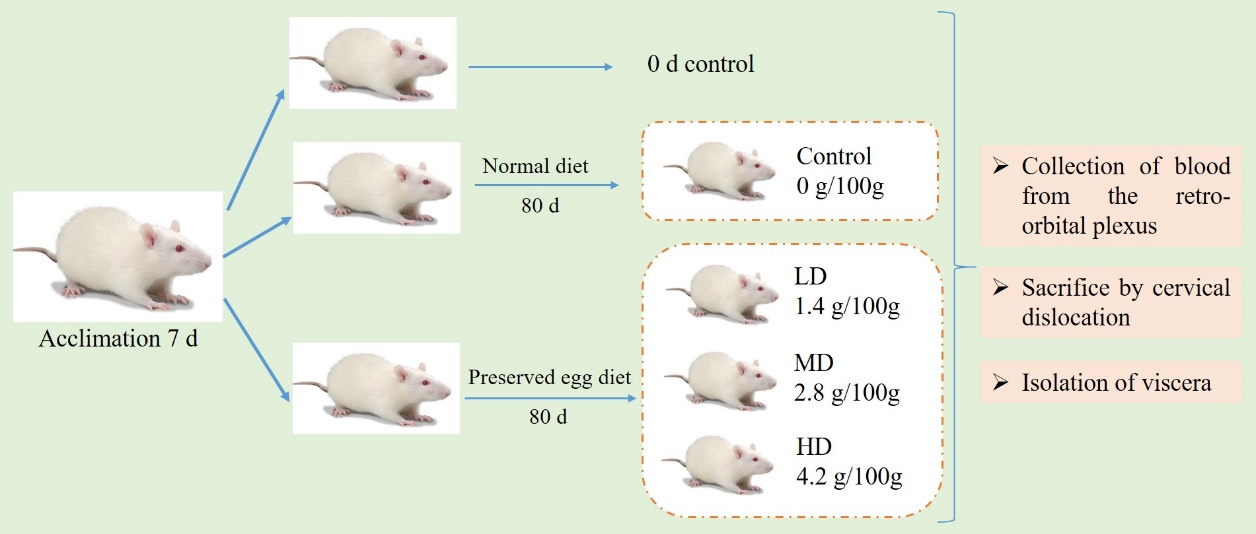
**Figure S.1. The design of the animal experimental groups.**

| Primers Names | GenBank accession no. | Primers Sequence |
| --- | --- | --- |
| Homo β-actin | NM_001101.5 | F: 5‘-ACTGGACTGTGGCATTGAGA-3’  R: 5‘-GCACAAAGCGACTGGATGAA-3’ |
| Homo caspase-3 | NM_001354781.2 | F: 5‘-ACATGCTGGCTTCGTTTCTG-3’  R: 5‘-TCTCAAGAGCACCGACATCA-3’ |
| Homo caspase-9 | NM_001229.5 | F: 5‘- GCCTTCTTTGAGTTCGGTGG-3’  R: 5‘-TCAGCCCAGACTCACATCAC-3’ |
| Homo CytC | NM_001177490.1 | F; 5‘-GGGTGATGTTGAGAAAGGCA-3’  R: 5‘-TCCCCAGATGATGCCTTTGT-3’ |

**Table S.1.** Sequence of primers in RT-qPCR
